# Aberrant Amplitude of Low-Frequency Fluctuation and Degree Centrality within the Default Mode Network in Patients with Vascular Mild Cognitive Impairment

**DOI:** 10.3390/brainsci11111534

**Published:** 2021-11-19

**Authors:** Haoyuan Li, Xiuqin Jia, Yingying Li, Xuejia Jia, Qi Yang

**Affiliations:** 1 Department of Radiology, Beijing Chaoyang Hospital, Capital Medical University, Beijing 100020, China; shucklelhy@mail.ccmu.edu.cn (H.L.); xiuqin.jia@mail.ccmu.edu.cn (X.J.); liyingying0625@mail.ccmu.edu.cn (Y.L.); jiaxuejia@mail.ccmu.edu.cn (X.J.); 2 Key Laboratory of Medical Engineering for Cardiovascular Disease, Ministry of Education, Beijing 100020, China; 3 Beijing Advanced Innovation Center for Big Data-Based Precision Medicine, Beijing 100020, China

**Keywords:** vascular mild cognitive impairment (VaMCI), amplitude of low-frequency fluctuation (ALFF), degree centrality (DC), gray matter (GM) atrophy, default mode network (DMN)

## Abstract

This study aimed to investigate whole-brain spontaneous activities changes in patients with vascular mild cognitive impairment (VaMCI), and to evaluate the relationships between these brain alterations and their neuropsychological assessments. Thirty-one patients with VaMCI and thirty-one healthy controls (HCs) underwent structural MRI and resting-state functional MRI (rs-fMRI) and neuropsychological assessments. The functional alterations were determined by the amplitude of low-frequency fluctuation (ALFF) and degree centrality (DC). The gray matter volume (GMV) changes were analyzed using voxel-based morphometry (VBM). Linear regression analysis was used to evaluate the relationships between the structural and functional changes of brain regions and neuropsychological assessments. The VaMCI group had significantly lower scores in the Montreal Cognitive Assessment (MoCA), and higher scores on the Hamilton Anxiety Rating Scale (HAMA) and Hamilton Depression Rating Scale (HAMD). Compared to the HCs, the VaMCI group exhibited GM atrophy in the right precentral gyrus (PreCG) and right inferior temporal gyrus (ITG). VaMCI patients further exhibited significantly decreased brain activity within the default mode network (DMN), including the bilateral precuneus (PCu), angular gyrus (AG), and medial frontal gyrus (medFG). Linear regression analysis revealed that the decreased ALFF was independently associated with lower MoCA scores, and the GM atrophy was independently associated with higher HAMD scores. The current finding suggested that aberrant spontaneous brain activity in the DMN might subserve as a potential biomarker of VaMCI, which may highlight the underlying mechanism of cognitive decline in cerebral small vessel disease.

## 1. Introduction

Vascular mild cognitive impairment (VaMCI) is the early stage of vascular dementia (VaD), considered to be the most critical subtype of vascular disease due to cerebral small vessel disease (CSVD) [1]. Early diagnosis and intervention of VaMCI are of particular importance. Understanding the mechanism of brain function of cognitively influenced VaMCI could help delay the progress and prevent the occurrence of dementia [2]. Even though several research studies have examined the neural mechanisms of CSVD and vascular cognitive impairment (VCI) during the last decade, the definitive conclusion of the neuropathological mechanism in VaMCI remains undetermined.

More advanced than traditional MRI methods that are used to diagnose pathological changes in the late stage of brain vascular disease, amplitude low-frequency fluctuation (ALFF) is a kind of non-invasive resting-state functional MRI (rs-fMRI) technique, measuring the spontaneous total power of the BOLD signal, reflecting regional spontaneous neuronal activity in an early stage of the disease [3]. Altered ALFF values in brain regions have been identified predominantly in the default mode network (DMN) in VaMCI [4,5,6]. However, these functional alterations within the DMN still remain inconsistent. For example, decreased ALFF in the bilateral medial prefrontal cortex (anterior part of DMN) and increased ALFF in the right posterior cingulate cortex (PCC)/precuneus (PCu) (posterior part of DMN) have been reported in VaMCI patients compared to healthy controls (HCs) [4]. By contrast, decreased ALFF in the posterior DMN has been reported in the posterior parietal cortex and PCu [5,6], and increased ALFF in the anterior DMN of the bilateral anterior cingulate cortex, superior medial frontal gyrus, and orbital frontal cortex has been identified in VaMCI compared to HCs [5]. Thus, the reliability of spontaneous brain activity, particularly in the DMN in patients with VaMCI still needs to be answered.

Furthermore, CSVD frequently experiences brain functional alteration accompanied by structural changes [7]. In particular, changes in gray matter volume (GMV) were previously reported in VaMCI [4,6,8,9,10,11]. These regions involve the cortical regions of the frontal and temporal cortex, as well as subcortical regions of the pons, thalamus, caudate, and (para)hippocampus. Consequently, these morphometric changes may interact with functional activity. However, some rs-fMRI studies have considered the influence of GMV on functional activity [4,6,11], while others have not [5]. On this basis, the morphometric changes might, to some extent, confound the previous functional changes. GMV alterations are reported to be linked to ALFF deficits in amnestic MCI, suggesting that brain structural and functional impairment might occur in CSVD patients with MCI [12]. In addition, GM atrophy is responsible for cognitive decline in VaMCI, such as memory loss, attention/executive dysfunction, language dysfunction, visuospatial function [9,10], and depression [13]. In the present study, to better understand the neural mechanisms underlying VaMCI, structural influence on functional alterations in VaMCI was considered.

Different from ALFF which reflects regional brain activities, degree centrality (DC) reflects the information flow in the global network connectivity at the voxel level, and provides insights into the relationship between the local activity and the global network [14]. Recently, the abnormality of DC has been widely reported in MCI, AD, and PD [8,9]. Therefore, combined with the ALFF and DC, we aimed to explore the aberrant spontaneous brain activities in patients with VaMCI compared to HCs and examine the relationship between these alterations and clinical characteristics. It was further hypothesized that abnormal spontaneous brain activity in the DMN might be exhibited in patients with VaMCI, independent of confounding factors.

## 2. Materials and Methods

### 2.1. Participants

For this study, 40 VaMCI patients and 36 HCs were recruited after excluding 9 patients with VaMCI and 5 HCs due to quality control of MRI images. Finally, 31 VaMCI patients (20 males, 62.87 ± 7.07 years old) and 31 HCs (14 males, 59.35 ± 8.15 years old) who were matched for age and gender were recruited in this study. The experimental procedure was approved by the Research Ethics Committee. Written informed consent was acquired from each participant after the study was fully explained.

The inclusion criteria for the VaMCI groups were derived from the clinical practice guideline for cognitive impairment of CSVD of China (2019) [1] and Diagnostic Criteria for Vascular Cognitive Disorders—A VASCOG Statement [15]: (1) presence of cognitive decline complaint by patient participant or caregiver, and presence of cognitive impairment that was not enough to affect life; (2) there exists evidence of MRI markers of small cerebrovascular lesions, including white matter lesions, lacunar infarcts, cerebral microbleeds, enlarged perivascular space, or cortical microinfarcts in MRI imaging; (3) a Clinical Dementia Rating Scale (CDR) score = 0.5; (4) cognitive activities have not reached the standard of a diagnosis of dementia in the Diagnostic and Statistical Manual of Mental Disorders, Fourth Edition (DSM-IV); and (5) Montreal Cognitive Assessment (MoCA) score < 26.

The exclusion criteria for the VaMCI group: (1) dementia or severe cognitive impairment; (2) no evidence of cerebrovascular lesions in MRI imaging; (3) history of a neurological or psychiatric disorder affecting cognition (e.g., large cerebrovascular disease, white matter disease, brain tumor, depression); and (4) contraindications for MRI, being a cardiac pacemaker user.

The inclusion criteria for the HC group: (1) without neurological or psychiatric diseases diagnosed currently or previously in physical examinations and neuropsychological tests, (2) no subjective or caregiver complaints of cognitive impairment, and (3) no abnormal findings on brain MRI.

All of the participants received standardized neuropsychological assessments. The global cognitive level was assessed by MoCA. Emotional performances were evaluated by the Hamilton Anxiety Rating Scale (HAMA) for anxiety and the Hamilton Depression Rating Scale (HAMD) for depression. Other examinations included Clinical Dementia Rating (CDR) and Activity of Daily Living (ADL). An ADL score ≤ 20 considers that the individual’s daily living activity was normal or corrected-to-normal status.

### 2.2. Image Data Acquisition

MRI imaging data were obtained using a 3.0 Tesla Scanner (Discovery MR750W, GE Healthcare, Chicago, IL, USA) and an 8-channel head coil. All participants were told to lie still and close their eyes, breathe quietly, and remain awake and motionless. Foam padding was applied to limit head motion, and earplugs were employed to reduce noise during scanning.

A T2-weighted image (T2WI), diffusion-weighted imaging (DWI), and fluid-attenuated inversion recovery (FLAIR) were acquired. Axial T2WI: repetition time (TR) = 4742 ms; echo time (TE) = 119 ms; slice thickness = 6 mm; field of view (FOV) = 240 × 240; matrix size = 416 × 416; and slice number = 19. Axial DWI: repetition time (TR) = 4880 ms; echo time (TE) = 77 ms; b-values = 0, 1000 s/mm^2^; slice thickness = 6 mm; field of view (FOV) = 240 × 240; matrix size = 130 × 160; and slice number = 38. Axial FLAIR: repetition time (TR) = 9000 ms; echo time (TE) = 93 ms; slice thickness = 6 mm; field of view (FOV) = 240 × 240; matrix size = 256 × 256; and slice number = 19.

High-resolution T1-weighted images of the whole brain were received using a sagittal 3D magnetization-prepared rapid gradient echo (MP-RAGE) sequence with the following parameters: repetition time (TR) = 8.464 ms; echo time (TE) = 3.248 ms; inversion time (TI) = 450 ms; slice thickness = 1 mm; flip angle = 12°; field of view (FOV) = 256 × 256; matrix size = 256 × 256; and slice number = 188. Rs-fMRI were acquired using an echo-planar imaging (EPI) sequence parameters: repetition time (TR) = 2000 ms; echo time (TE) = 30 ms; slice thickness = 3.6 mm; gap = 0.4 mm; flip angle = 90°; field of view (FOV) = 220 × 220; matrix size = 64 × 64; and slice number = 36.

### 2.3. MRI Data Processing

Rs-fMRI data were pre-processed and analyzed using the data processing assistant for rs-fMRI (DPARSF 5.0 http://www.rfmri.org, accessed on 8 October 2020; [16]), and statistical parametric mapping 12 packages (SPM12 http://www.fil.ion.ucl.ac.uk/spm, accessed on 8 October 2020). The first 10 functional images volumes were removed to reduce the fluctuation of MRI signals. The remaining images of each subject have all layers corrected for slice timing to reduce the within-scan acquisition time differences between slices. Then images of each subject were realigned to reduce the influence of head motion during the scan. The time slices were scrubbed if the head motion exceeded 2 mm in displacement (x, y, or z) or >2° in rotation. Then, the realigned images were co-registered to T1 images (through DARTEL). T1 images were segmented into GM, white matter (WM), and cerebrospinal fluid (CSF). Then the co-registered images were spatially normalized into Montreal Neurological Institute (MNI) space using transformations from segmentation and resampled them to 3 × 3 × 3 mm^3^ voxels. The functional images were smoothed with a 6 mm full width at a half-maximum (FWHM) isotropic Gaussian kernel. A linear nuisance covariates regression was performed to remove the interference of WM and CSF signals. Detrend and filter were used to reduce higher frequency noise and the lower frequency drift caused by physiological interference.

The time series were transformed to the frequency domain using the fast Fourier transform (FFT) algorithm, and the power spectrum was obtained by square-rooted FFT and averaged across 0.01–0.08 Hz at each voxel. To reduce the global effects of variability among the subjects, the mean ALFF was taken as the ALFF of each voxel divided within the whole-brain mask obtained previously. The global mean ALFF was calculated within the brain. The background and other tissues outside the brain were removed. Finally, the mean ALFF values of each brain region of each significant cluster were extracted.

DC is a graph theory-based approach to calculate the temporal correlation between a voxel and all other brain voxels within the mask at the voxel-wise level [14]. After preprocessed data, we acquired an *n × n* matrix of Pearson’s correlation coefficients between any pair of voxels, where *n* is the voxel number of the GM mask. The matrix of Pearson’s correlation coefficients was set at the threshold *r* > 0.25 for computing the GM voxel. Only positive Pearson’s correlation coefficients were considered. Binarized global mean DC values of the whole-brain network were calculated as the sum of the significant connections in a given voxel at the individual level.

A voxel-based morphometry (VBM) analysis was acquired to explore the structural alteration in VaMCI. Using the DPARSF software, 3D-T1-weighted images examined GMV alterations in VaMCI and HCs with VBM analysis. All T1-weighted images were segmented into GM, WM, and CSF. Then, they were normalized to the MNI template. Finally, they were smoothed with an 8 mm FWHM isotropic Gaussian kernel. Total intracranial volume (TIV) was used as a covariate in the GMV analysis.

### 2.4. Statistical Analysis

The statistical software package SPSS 22.0 was used in statistical analysis, including comparing demographic, neuropsychological scores, and extracted structural and functional values between the VaMCI and HCs. The Kolmogorov–Smirnov test was used to test normality for selecting parametric tests or non-parametric tests. The Chi-square test was used to compare gender differences between groups. The two-sample *t*-test for the parametric test and Mann–Whitney test for the non-parametric test were adopted. Statistical significance was set at *p* < 0.05.

Voxel-wise two-sample *t*-tests were performed to detect the group differences in ALFF, DC values, and GMV values, controlled for age, gender, and education years. To further exclude the effect of structural change on functional change, GMV was used as a covariate. ALFF and GMV results were thresholded at a voxel-wise *p* < 0.001 (uncorrected) combined with a cluster-wise *p* < 0.05 (FWE corrected). DC results were thresholded at a voxel-wise *p* < 0.005 (uncorrected) combined with a cluster-wise *p* < 0.05 (FWE corrected). The XjView software (http://www.alivelearn.net/xjview, accessed on 23 October 2020) was used to report the brain anatomical regions.

Regions with significant alterations in patients were further defined as regions of interest (ROIs). To explore the relationship between values in these ROIs and patients’ performance on neuropsychological assessments, partial correlations were then performed after adjusting for age, sex, education level, and GMV changes as nuisance variables of no interest. Multiple linear regression was analyzed for MoCA, HAMA, and HAMD as a dependent variable, while age, gender, educational levels, and extracted mean values of ALFF, DC, and GMV were analyzed as variables. The forward method was adopted. A composite *z*-score of ALFF, DC, and GMV values was calculated.

## 3. Results

### 3.1. Demographic and Neuropsychological Tests Results

The details of the demographic characteristics and neuropsychological tests scores of all the participants are shown in Table 1. There was no significant difference in age and gender between the two groups. There was a significant difference between the two groups in education level, MoCA, HAMA, and HAMD. Compared to the HC group, the VaMCI group had significantly lower education level (*p* = 0.017) and MoCA scores (*p* < 0.001), and significantly higher HAMA scores (*p* = 0.009) and HAMD scores (*p* = 0.006).

### 3.2. VBM Results

Compared to the HC group, the VaMCI group showed a significant GMV decrease in the right precentral gyrus (PreCG) (MNI coordinate: 36, −20, 63, with 470 voxels, *t* = 4.42) and right inferior temporal gyrus (ITG) (MNI coordinate: 50, −21, −27, with 383 voxels, *t* = 4.94) (Supplementary Figure S1A,B). These regions also survived adjusting for age, gender, and education level (voxel-wise threshold *p* < 0.001).

### 3.3. ALFF Results

**Figure 1:**
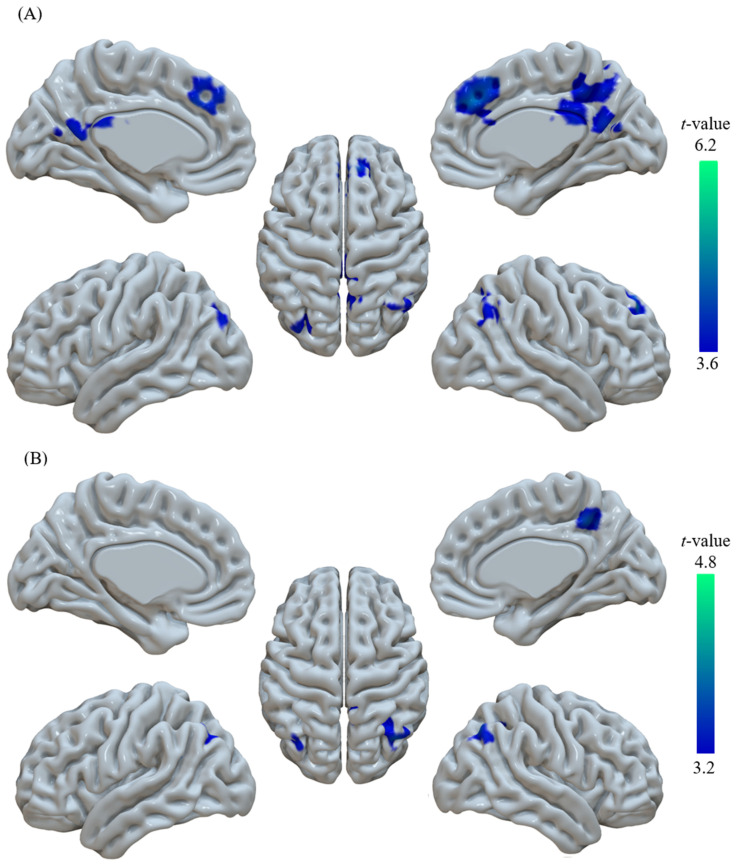
Altered brain regions in the VaMCI group compared with HCs. (**A**) ALFF decrease in the bilateral PCu, R.AG, and R.medFG in the VaMCI group. (**B**) DC decrease in the R.PCu and bilateral AG in the VaMCI group. Color bars indicate *t*-values. PCu, precuneus; AG, angular gyrus; medFG, medial frontal gyrus; L., left; R., right.

**Figure 2:**
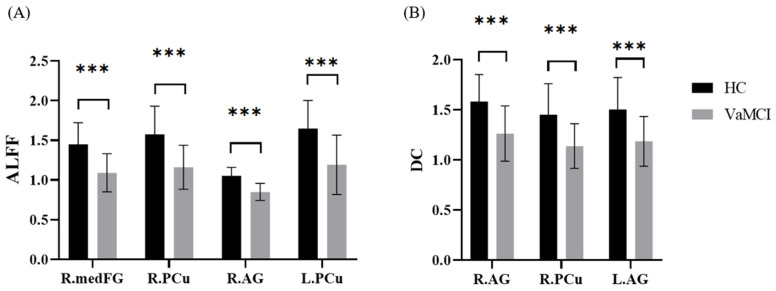
Comparison of significant brain regions between VaMCI group and HC group in extracted ALFF and DC values. (**A**) Comparison of ALFF values in the bilateral PCu, R.AG, and R.medFG between the VaMCI group and HC group. (**B**) Comparison of DC values in the R.PCu and bilateral AG between the VaMCI group and HC group. HC, healthy control; VaMCI, vascular mild cognitive impairment; PCu, precuneus; AG, angular gyrus; medFG, medial frontal gyrus; L., left; R., right. *** *p* < 0.001.

Compared to the HC group, the VaMCI group showed a significant ALFF decrease in the DMN, including the right precuneus (PCu), right angular gyrus (AG), right medial frontal gyrus (medFG), and left PCu (Table 2, Figure 1A and Figure 2A), which remained after controlling for the nuisance variables of age, gender, education years, GMV, and HAMD.

### 3.4. DC Results

Compared to the HC group, the VaMCI group showed a significant DC decrease in the DMN, including the right AG, right PCu, and left AG (Table 3, Figure 1B and Figure 2B). After we used GMV as a covariate, the DC values pattern remained highly similar to those without a covariate.

### 3.5. Linear Regression Analysis

**Figure 3:**
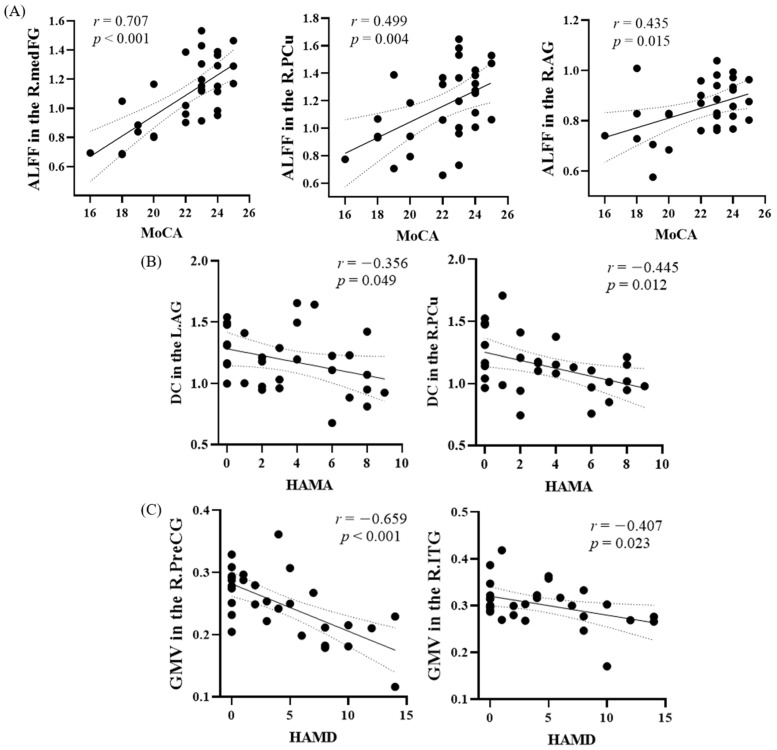
Linear correlation between brain functional and structural changes and clinical characteristics in VaMCI. (**A**) Positive correlation between MoCA and ALFF in the R.medFG, R.PCu, and R.AG. (**B**) Negative correlation between HAMA and DC in the R.PCu and L.AG. (**C**) Negative correlation between HAMD and GMV in the R.PreCG and R.ITG. PCu, precuneus; AG, angular gyrus; medFG, medial frontal gyrus; PreCG, precentral gyrus; ITG, inferior temporal gyrus; L., left; R., right.

Mean ALFF, DC, and GMV values were extracted for each significant cluster of brain regions. Significantly positive correlations were found between MoCA scores and ALFF in the right medFG (*r* = 0.707, *p* < 0.001), right PCu (*r* = 0.499, *p* = 0.004), and right AG (*r* = 0.435, *p* = 0.015) in the VaMCI group (Figure 3A). Significantly negative correlations were found between HAMA scores and DC in the right PCu (*r* = −0.445, *p* = 0.012) and left AG (*r* = −0.356, *p* = 0.049) (Figure 3B), and between HAMD scores and GMV in the right PreCG (*r* = −0.659, *p* < 0.001) and right ITG (*r* = −0.407, *p* = 0.023) in the VaMCI group (Figure 3C). Furthermore, multiple linear regression analysis found that lower MoCA was independently associated with decreased ALFF scores (*p* < 0.001), DC was independently associated with higher HAMA scores (*p* = 0.048), and the GM atrophy was independently associated with higher HAMD scores (*p* < 0.001) (Table 4).

## 4. Discussion

In this study, we investigated whole-brain spontaneous activities in VaMCI patients and evaluated the relationships between the alterations of brain regions and cognitive and emotional neuropsychological indexes. The current study found that the VaMCI group exhibited altered brain activities in the DMN, particularly in the bilateral PCu, AG, and medFG, independent of GM atrophy. Further, the decreased ALFF was independently associated with lower cognitive decline measured by MoCA scores, while the changes of DC values in the DMN and GM atrophy contributed to emotional performance measured by the HAMA and HAMD tests, respectively.

Consistent with previous studies [4], the current findings indicated aberrant spontaneous brain activities within the DMN during resting state, specifically in the hub regions of the bilateral medFG and left PCC in VaMCI. DMN is a large-scale brain network comprised of highly connected cortical regions integrated with self-referential function, emotion, and episodic memory retrieval [17,18], exhibiting metabolically active decreases during attention-demanding tasks, but activity increases at rest [19]. In the current study, we noted that spontaneous brain activities decreased in both anterior and posterior parts of the DMN, especially in bilateral PCu, right AG, and right medFG. The PCu plays an important role mainly in visuospatial imagery, episodic memory retrieval, and self-processing operations [20]; the AG plays an important role in word reading, number processing, comprehension, attention, and spatial awareness [21]; the medFG plays an important role in decision making, discrimination, computation, and reasoning [22]. The reduced ALFF activities in the bilateral PCu, right AG, and right medFG may indicate DMN impairment in patients with VaMCI, suggesting global cognitive impairments. Thus, in our study, global cognitive impairments of VaMCI patients might be attributed to the aberrant activities in these DMN regions.

Previous study exhibited that ALFF decreased specifically in the anterior part of the DMN (i.e., the medFG) and increased in the posterior part of the DMN (i.e., the PCC/PCu) in VaMCI patients [4]. While another study showed increased ITG in the anterior part of DMN and decreased ALFF in the PCC/PCu, the posterior part of DMN [6]. The inconsistent results in DMN may be due to the different analysis approaches. In this study, we included age, gender, education level, and GMV as covariates in ALFF analysis to further control for their potential confounding effect. Moreover, in line with previous studies [23,24], the decreased ALFF in the DMN was positively correlated with MoCA scores in VaMCI, which suggested that the aberrant spontaneous brain activities was associated with cognitive decline in CSVD. In addition, a recent study has reported decreased regional homogeneity (Reho) and functional connectivity are associated with MoCA scores in VaMCI patients [25], suggesting that different fMRI measurements may provide mutual information about aberrant functional activities.

In this study, the significantly decreased DC regions in DMN overlapped with the ALFF, including bilateral AG and right PCu in VaMCI compared to HCs, consistent with a previous study [4], which exhibited decreased functional connectivity density in the PCC/PCu, the medFG, and the middle temporal gyrus. In addition, VaMCI patients exhibited a significant correlation between HAMA and decreased DC. Furthermore, HAMD scores increased in VaMCI patients, indicating that VaMCI patients might be more prone to have depression symptoms. Evidence suggests that depression plays a critical role in the pathogenesis of cognitive impairment and cerebrovascular events [26,27,28,29].

Previous studies suggest that CSVD is accompanied by structural changes such as cortical thinning and GM atrophy due to the remote effect of the white matter hypertension (WMH) and infarct [7,30], and the WMH lesions are able to affect cognition impairments [31]. Widespread GM atrophy has been reported in many cortical and subcortical regions, such as frontal and temporal cortex, pons, cerebellum, thalamus, hippocampus, parahippocampal gyrus, and caudate in VaMCI patients [4,8,9]. In the current study, decreased GMV was identified in the right PreCG and right ITG in VaMCI patients. PreCG is the main brain area of the primary motor cortex, which plays an important role in motor control [32]. At the same time, ITG plays a role in visual recognition, decision making, and lexical/phonologic decisions [33]. A previous study found that structural network disruption can damage mood regulation circuits and cause depressive symptoms [29]. On this basis, the current study also identified that GM atrophy in PreCG and ITG were negatively correlated with HAMD scores.

Some limitations should be acknowledged in this study. First, the sample size of participants was relatively small. In order to obtain a more reliable result, large sample size is required in further studies. Second, to measure the cognitive decline in specific cognitive domains, comprehensive neuropsychological assessments should be performed. Third, to explore the causal relationship between brain changes and the progression of this disease, a longitudinal study is needed in the future. Fourth, due to the lack of clinical data in our study, the conditions of blood glucose variations, aortic macrovascular disease, and ApoEε4 were not clear. Further study should exclude the impact of these potential confounding factors. Fifth, to better understand the relationship between brain functional and structural alterations, the B-matrix spatial distribution method DTI (BSD-DTI), which is able to decrease scanning time, maintain a minimal number of diffusion gradient directions and high resolution, and reduce spatial systematic errors in tractography by gradients inhomogeneity [34,35,36], should be considered in future experiments.

## 5. Conclusions

The current findings suggested that aberrant spontaneous brain activity in the DMN might subserve as a potential biomarker of VaMCI, which may highlight the underlying mechanism of cognitive decline in cerebral small vessel disease.

## Figures and Tables

**Table 1:** Demographic and clinical characteristics.

	HC (*N* = 31)	VaMCI (*N* = 31)	*p*-Value
Age (years)	59.35 (8.15)	62.87 (7.07)	0.075
Gender (male/female)	14/17	20/11	0.446
Education level (years)	11(9, 12)	9 (8, 11)	0.017
MoCA score	28 (26, 30)	23(20, 24)	<0.001
HAMD score	0 (0, 1)	3 (0, 8)	0.006
HAMA score	0 (0, 3)	3 (0, 6)	0.009

Normal distribution data are presented as means (SD). Non-normal distribution data are presented as median (the first quartile, the third quartile). Gender data were analyzed with Chi-square test; age was analyzed with two-sample *t*-test; education level, MoCAMMSE, HAMD, and HAMA were analyzed with the Mann–Whitney test. HC, healthy control; VaMCI, vascular mild cognitive impairment; MoCA, Montreal Cognitive Assessment; HAMA, Hamilton Anxiety Rating scale; HAMD, Hamilton Depression Rating scale.

**Table 2:** Decreased ALFF in VaMCI compared to HC.

Region	Cluster Size	MNI Coordinate	*t*-Value
	(voxel)	(x, y, z)	
R.PCu	258	(15, −45, 45)	5.05
R.AG	125	(54, −63, 45)	4.91
R.medFG	58	(6, 39, 36)	6.08
L.PCu	44	(−33, −81, 39)	4.35

Results were thresholded at a voxel-wise *p* < 0.001 (uncorrected) combined with a cluster-wise *p* < 0.05 (FWE corrected). PCu, precuneus; AG, angular gyrus; medFG, medial frontal gyrus; L., left; R., right; MNI, Montreal Neurological Institute.

**Table 3:** Decreased DC in VaMCI compared to HC.

Region	Cluster Size	MNI Coordinate	*t*-Value
	(voxel)	(x, y, z)	
L.AG	258	(−36, −60, 33)	4.15
R.PCu	224	(6, −45, 42)	4.24
R.AG	220	(42, −72, 48)	4.69

Results were thresholded at a voxel-wise *p* < 0.005 (uncorrected) combined with a cluster-wise *p* < 0.05 (FWE corrected). PCu, precuneus; AG, angular gyrus; L., left; R., right; MNI, Montreal Neurological Institute.

**Table 4:** Linear regression between MoCA and ALFF, HAMA and DC, and HAMD and GMV.

		Unstandardized Coefficient		Standardized Coefficient	*t*	*p*
		*B*	*SE(B)*	*β*		
MoCA	Constant	23.528	0.488		48.193	<0.001 ***
	Age	−0.160			−1.103	0.280
	Gender	0.006			0.042	0.967
	Edu. level	0.043			0.292	0.773
	GMV	0.092			0.627	0.536
	ALFF	2.122	0.489	0.627	4.338	<0.001 ***
	DC	−0.077			−0.476	0.638
Registered R square: 0.373; ANOVA: <0.001						
HAMA	Constant	1.727	0.836		2.065	0.048 *
	Age	0.224			1.249	0.222
	Gender	2.773	1.041	0.443	2.663	0.013 *
	Edu. level	0.065			0.368	0.716
	GMV	−0.323			−2.043	0.051
	ALFF	0.050			0.292	0.773
	DC	−0.237			−1.349	0.188
Registered R square: 0.169; ANOVA: 0.013						
HAMD	Constant	0.220	1.104		0.199	0.844
	Age	0.083			0.517	0.610
	Gender	2.963	1.251	0.322	2.368	0.025 *
	Edu. level	−0.084			−0.589	0.561
	GMV	−3.426	0.735	−0.633	−4.662	<0.001 ***
	ALFF	−0.086			−0.615	0.544
	DC	−0.114			−0.775	0.445
Registered R square: 0.448; ANOVA: <0.001						

Edu., education. ANOVA, analysis of variance * *p* < 0.05, *** *p* < 0.001.

## Data Availability

The data presented in this study are available on request from the corresponding author. The data are not publicly available due to data privacy.

## Supplementary Materials

The following are available online at https://www.mdpi.com/article/10.3390/brainsci11111534/s1, including other results and findings that are not included in the main contents. Figure S1: Altered brain regions of GMV in the VaMCI group compared with HCs. (A) GM atrophy in the R.PreCG and R.ITG in the VaMCI group. Results were thresholded at a voxel-wise *p* < 0.001 (uncorrected) combined with a cluster-wise *p* < 0.05 (FWE corrected). Color bar indicates the *t*-value. (B) Comparison of significant brain regions between the VaMCI group and HC group of the extracted GMV values in the R.PreCG and R.ITG. PreCG, precentral gyrus; ITG, inferior temporal gyrus; L., left; R., right. *** *p* < 0.001.
