# Supplementary material for: Aberrant Amplitude of Low-Frequency Fluctuation and Degree Centrality within the Default Mode Network in Patients with Vascular Mild Cognitive Impairment
Source: Brain Sci. 2021 Nov 19;11(11):1534. doi: 10.3390/brainsci11111534 (PMC8615791; doi:10.3390/brainsci11111534)
Supplement: Supplementary file 1 [file brainsci-11-01534-s001.zip › brainsci-1412198-supplementary.pdf]

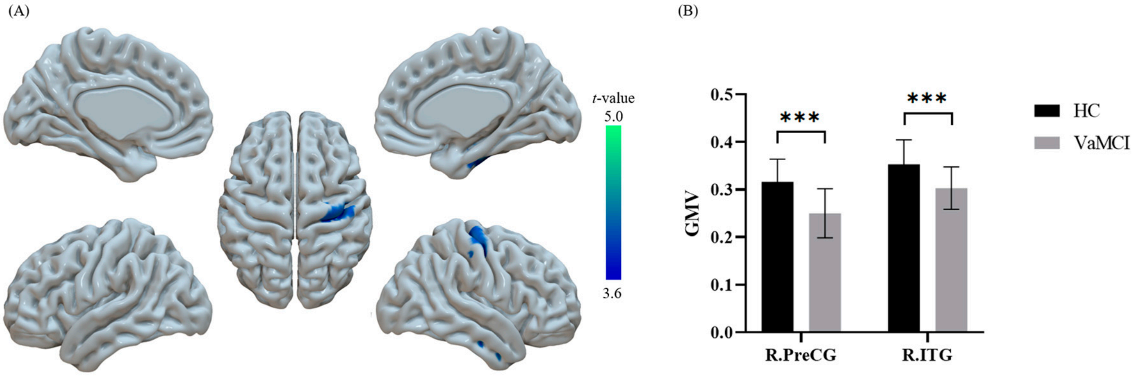


**Figure S1.** Altered brain regions of GMV in the VaMCI group compared with HCs. (**A**) GM. atrophy in the R.PreCG and R.ITG in the VaMCI group. Results were thresholded at a voxel-wise $p < 0.001$ (uncorrected) combined with a cluster-wise $p < 0.05$ (FWE corrected). Color bar indicates the *t*-value. (**B**) Comparison of significant brain regions between the VaMCI group and HC group of the extracted GMV values in the R.PreCG and R.ITG. PreCG, precentral gyrus; ITG, inferior temporal. gyrus; L., left; R., right. *** $p < 0.001$.
